# Supplementary material for: Thermo-adaptive interfacial solar evaporation enhanced by dynamic water gating
Source: Nat Commun. 2024 Jul 22;15:6157. doi: 10.1038/s41467-024-50279-z (PMC11263690; doi:10.1038/s41467-024-50279-z)
Supplement: Supplementary file 1 — Supplementary Information [file 41467_2024_50279_MOESM1_ESM.pdf]

Supplementary Information

Thermo-Adaptive Interfacial Solar Evaporation Enhanced by Dynamic Water Gating

Yi Wang [1,2 ^*], Weinan Zhao [1^], Yebin Lee [1], Yuning Li [1], Zuankai Wang [2], Kam Chiu Tam [1*]

[1] Department of Chemical Engineering, Waterloo Institute for Nanotechnology, University of Waterloo, 200 University Avenue West, Waterloo, Ontario, Canada N2L 3G1

[2] Department of Mechanical Engineering, The Hong Kong Polytechnic University, Hong Kong, China.

^ These authors contributed equally

Outline

Supplementary Methods: Page 3-4

Supplementary Notes: Page 5-7

Supplementary Figures: Page 8-25

Supplementary Tables: Page 26-27

Supplementary References: Page 28-29

Supplementary Method 1. DSC Measurement of Vaporization Enthalpy

We measured the evaporation enthalpy of water in evaporators using differential scanning calorimetry. The vaporization enthalpy measurements were carried out by placing the saturated foam in an open aluminum crucible and subjecting it to a linear heating rate of 5 K min^{-1} under a nitrogen flow rate of 20 mL min^{-1}, within a temperature range of 20 to 180 °C.

Supplementary Method 2. Lithium-ion Solvation Experiment

We prepared a LiCl water solution varying the concentration in 50 g L^{-1} and 100 g L^{-1}, and the evaporated vapor was condensed and collected. The Li^+ concentration in the collected water was measured using Inductively Coupled Plasma Mass Spectrometry (ICP-MS). To verify the formation of water clusters during evaporation, we utilized the solvation properties of Lithium-ion (Li^+) in a water collection experiment, demonstrating enhanced evaporation through water cluster formation.

Supplementary Note 1. Surface Free Energy Calculation

Surface free energy (γ) was composed of various inter-molecular attractive forces, including polar components (γ_s^p) and dispersive components (γ_s^d) at the interface. Based on the contact angle data and fitted according to the Owens, Wendt, Rabel and Kaelble (OWRK) method[1], the γ was calculated using the following equation (1):

$$1+\cos\theta=2\sqrt{\gamma_s^d}\left(\frac{\sqrt{\gamma_l^d}}{\gamma_{lv}}\right)+2\sqrt{\gamma_s^p}\left(\frac{\sqrt{\gamma_l^p}}{\gamma_{lv}}\right) \quad (1)$$

where θ was the contact angle of liquid, the superscripts p and d corresponding to the polar and dispersion force components, respectively. γ_{lv} was the free energy between liquid and solid against their saturated vapor, while γ_l^d and γ_l^p referred to the free energies of liquids as reported in many references[2,3].

Supplementary Note 2. Energy conversion efficiency

The solar energy conversion efficiency (η) is calculated by the equation:

$\eta = m h_v / C_{opt} P_0$ (2)

where m represents the net evaporation rate calculated by subtracting evaporation rate under dark condition from evaporation rate under one sun irradiation, hv refers to the equivalent evaporation enthalpy of water in SDWEs. P_0 is the solar irradiation power of one sun (1 kW m^{-2}). C_{opt} refers to the optical concentration on evaporator surface.

For p-SDWE, m = 3.58 – 0.45 = 3.13 kg m^{-2} h^{-1}, E_{eq} = 1080 J g^{-1}. Therefore, the solar energy conversion efficiency is 93.9% under one sun illumination.

Supplementary Note 3. Energy loss analysis

The energy loss during the entire process can be attributed to four main factors: conduction, radiation, convection, and reflection. Each heat loss parameters were calculated as follows:

(1) Conduction

$Q_{conduction} = CmD_T$

where $Q_{conduction}$ referred to the heat energy transfer from the evaporator to bulk water, *C* was the specific heat capacity of pure water (4.2 kJ kg^{-1} K^{-1}), m was the weight of water and D_T is the temperature change of bulk water under one sun irradiation within 3600 s. Therefore, the conduction heat loss of the evaporator was ~1.86% under 1 sun irradiation.

(2) Radiation

$Q_{radiation} = \varepsilon A\sigma(T_s^4 - T_0^4)$

where ε corresponding to the emissivity (assumed to be 1), A was the evaporator area (5.0 x 5.0 cm^2), and σ was the Stefan-Boltzmann constant (5.669×10^{-8} W m^{-2} K^{-4}), T_s was associated to the temperature of evaporator surface, T_0 was the ambient water temperature. The surface radiation heat loss accounts for ~2.84% of the total received energy.

(3) Convection

$Q_{convection} = hA\,(T_s - T_0)$

where h was the convection heat transfer coefficient (5 W m^{-2} K^{-1}). The convection heat loss made up for 1.9% of the total received energy.

(4) Reflection

The measured average reflection loss of p-DSF over the broad solar spectrum (200–2500 nm) was ~5.6%.

Therefore, the sum of calculated energy losses is 12.2% under 1 sun irradiation.

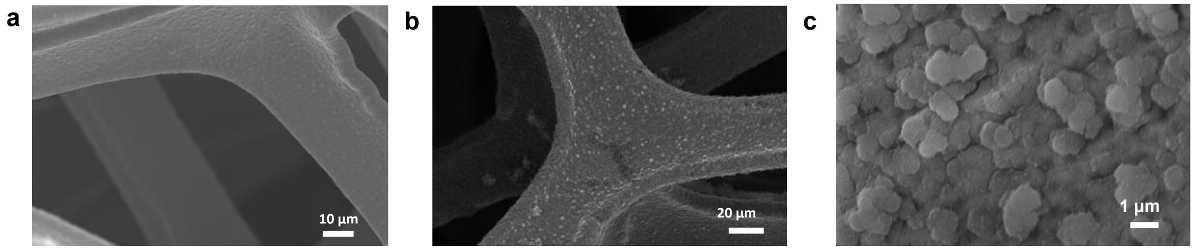

Supplementary Figure 1. The morphology of PDA layer: **a.** s-PDA, **b.** r-PDA, **c.** The PDA cluster on r-PDA layer.

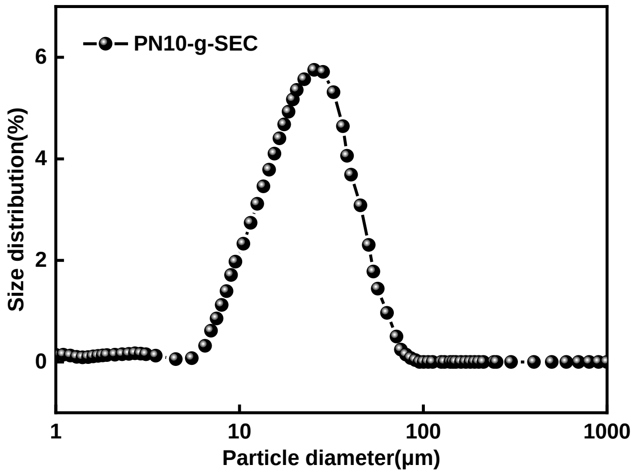

Supplementary Figure 2. Particle Size distribution of PN10-g-SEC.

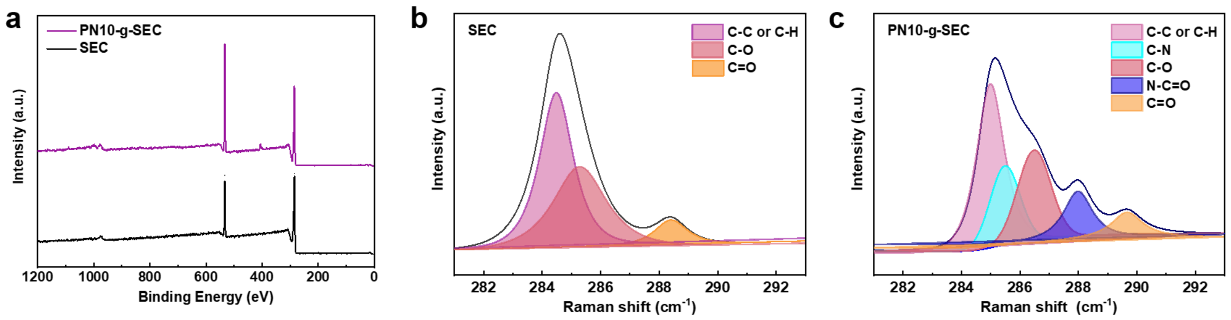

Supplementary Figure 3. XPS spectrum of PN10-g-SEC: **a.** XPS characterization of PN10-g-SEC and SEC, **b.** C1s fitting spectra of SEC, **c.** C1s fitting spectra of PN10-g-SEC

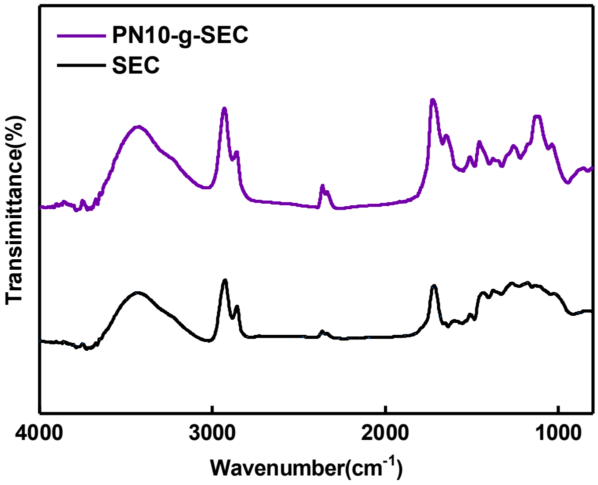

Supplementary Figure 4. FT-IR spectrum of SEC and PN10-g-SEC.

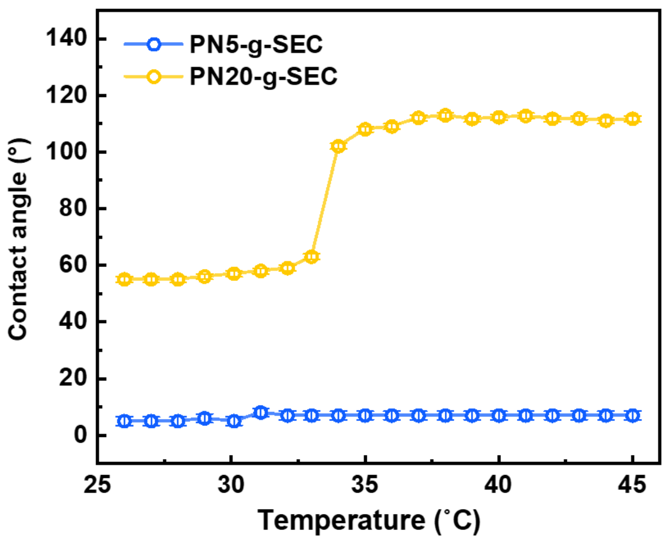

Supplementary Figure 5. Contact angle variation of PN5-g-SEC and PN20-g-SEC under different temperature

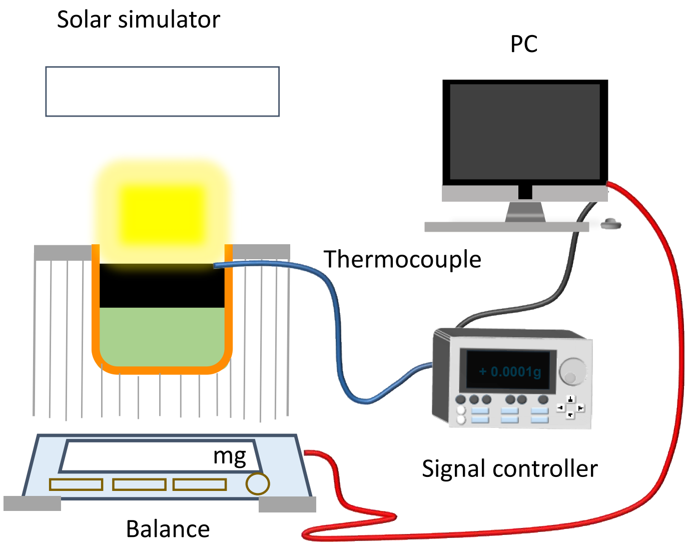

Supplementary Figure 6. The set-up for measuring the solar evaporation performance

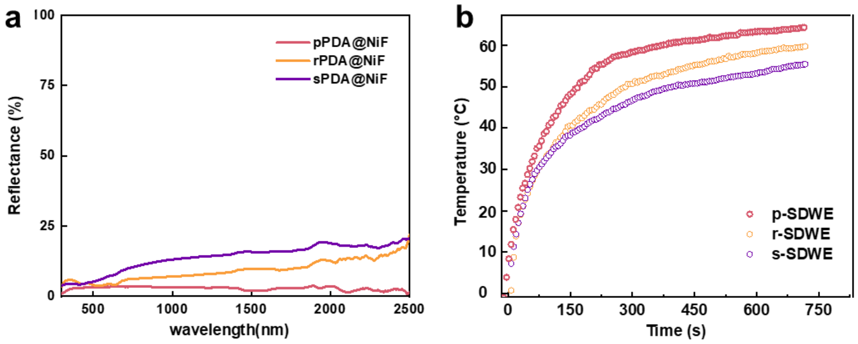

Supplementary Figure 7. a. Reflectance of PDA layer, compromising of pPDA@NiF, rPDA@NiF, sPDA@NiF, **b.** The surface temperature of dried SDWEs under solar illumination of 1KW m^{-}

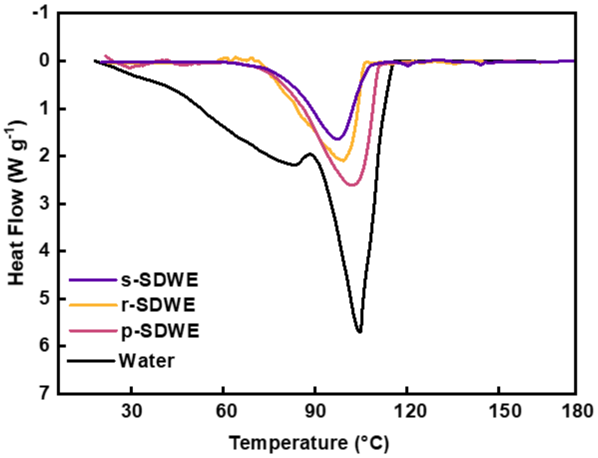

Supplementary Figure 8. Thermograms of pure water and SDWEs.

The effective specific heat capacity was calculated by comparing the heat flow of the measured samples with that of the standard sample. As shown in Supplementary Figure 8, a sharp peak is observed in pure water, followed by a significant decrease in the heat flow signal, indicating immediate water evaporation. However, the heat flow signals were differed in all the tested DSF samples, the peaks are much broader and lower than that of pure water.

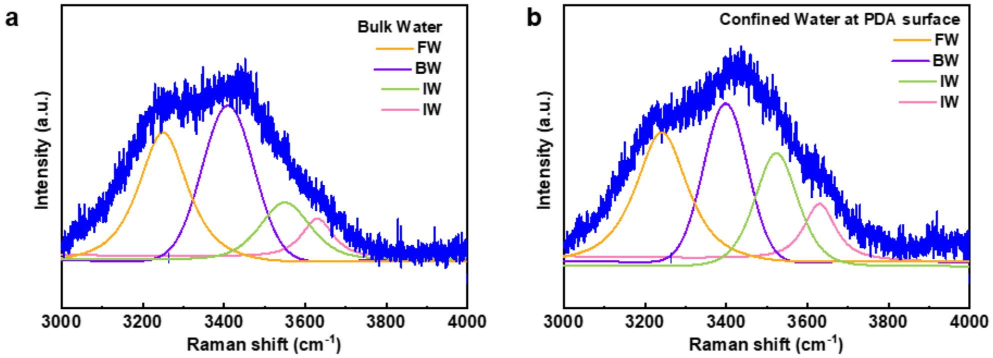

Supplementary Figure 9. Raman Spectroscopy of Water Structure: **a.** Bulk water, **b.** PDA coated surface.

To elucidate the variation in hydrogen bonding between bulk water and the confined thin water layer on polymeric materials, we analyzed the Raman Spectra of hydrogen bond vibrations. Peaks at 3250, 3410, 3520, and 3630 cm^{-1}, as displayed in Supplementary Figure 9, correspond to distinct water molecule types, aligning with reported literature. Compared to bulk water, the PDA surface exhibits a lower molar ratio of free water (FW), indicated by the peak at 3250 cm^{-1}. The affinity of water with the surface increases the ratio of bound water (BW) at 3410 cm^{-1}. Furthermore, the presence of intermediate water (IW), associated with peaks at 3520 and 3630 cm^{-1}, is significantly higher on the PDA surface. These findings suggest that the confined water within the PDA-assembled photothermal layer disrupts the stable hydrogen bonding network found in bulk water, thereby enhancing water evaporation efficiency.

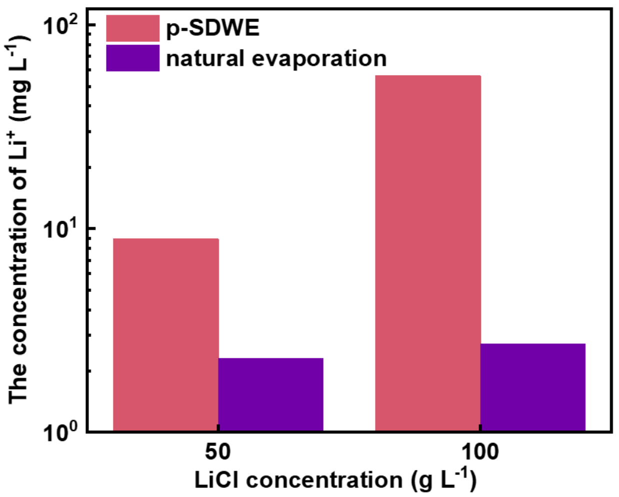

Supplementary Figure 10. The concentration of Li^+ collected from evaporation by p-SDWE and natural evaporation.

The collected water from our SDWE-based evaporation showed a Li^+ concentration of approximately 8.9 mg L^{-1} after evaporated 50 g L^{-1} and 56.3 mg L^{-1} after evaporated 50 g L^{-1}, while water from natural evaporation methods showed a much lower Li^+ concentration. These findings suggest that water evaporates in clusters from the SDWE, likely due to the strong solvation effect of Li^+. This conclusion, in conjunction with the Raman spectra analysis in Supplementary Figure 10 and the reduced latent heat detailed in Supplementary Table 1, supports the hypothesis that the existence of a thin water layer, enhanced by the surface characteristics of polymeric materials such as PDA, plays a significant role in boosting evaporation efficiency.

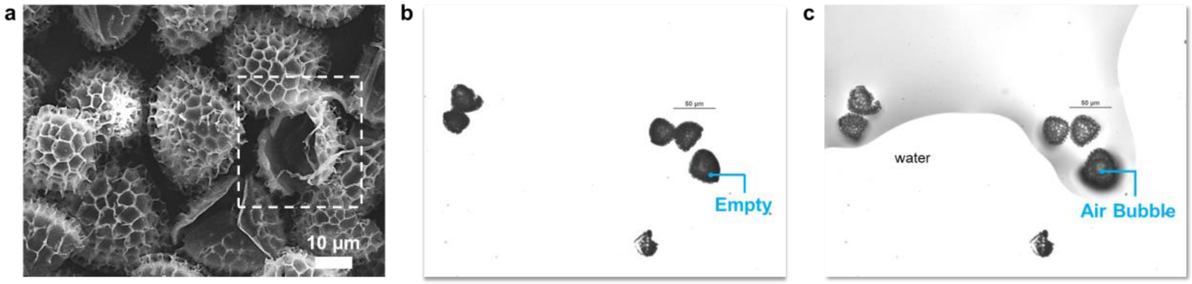

Supplementary Figure 11. a. SEM image of SEC, b. Optical microscopic image of SEC, c. Optical microscopic image of SEC immersed in water.

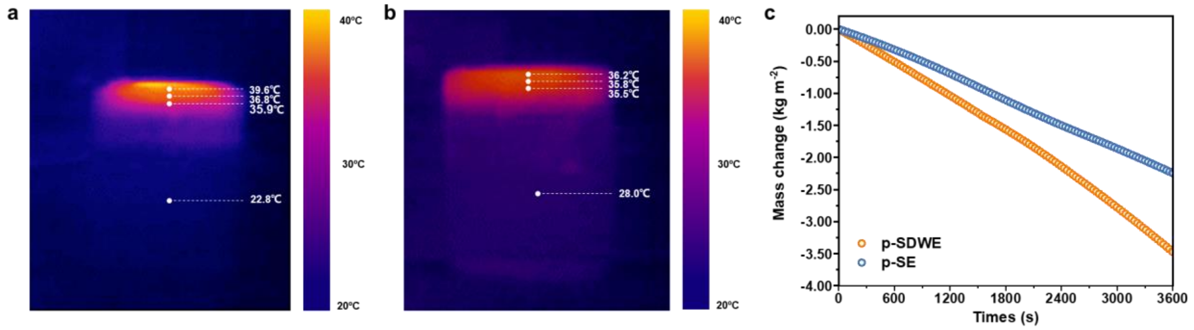

Supplementary Figure 12. The temperature distribution of the evaporator: a. p-SDWE, b. p-SE, as a reference sample, where the bottom without SEC layer, c. Mass change of evaporated water over time of the p-SDWE and the reference sample p-SE (without the SEC modification) under one sun solar illumination.

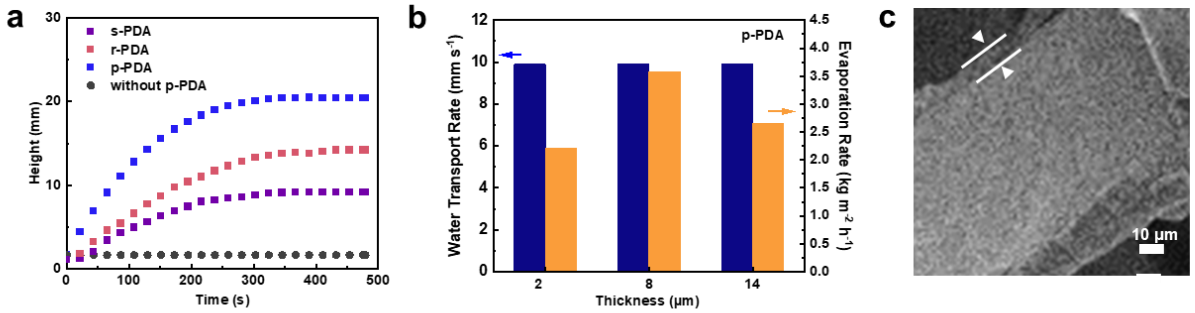

Supplementary Figure 13. a. Water transport rate along PDA layer varying different structures **b.** Illustration of the water transport rate and evaporation rate varying the thickness of PDA layer, **c.** The thickness of p-PDA layer on PN10-g-SEC

,

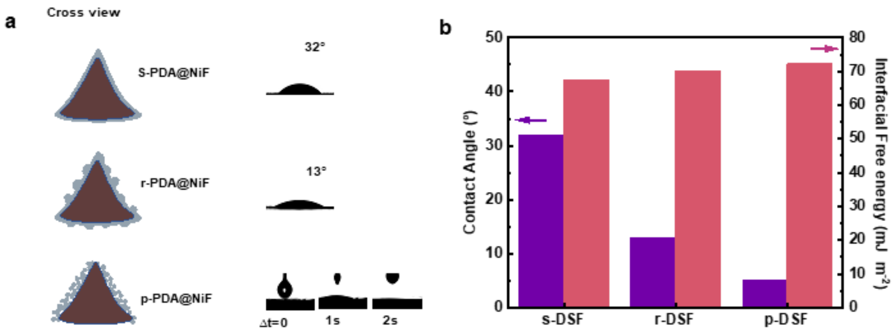

Supplementary Figure 14. a. Ilustration of water affinity and its corresponding contact angles of PDA layer varying the structure, **b.** Contact angle and interfacial free energy of s-PDA, r-PDA and p-PDA layer.

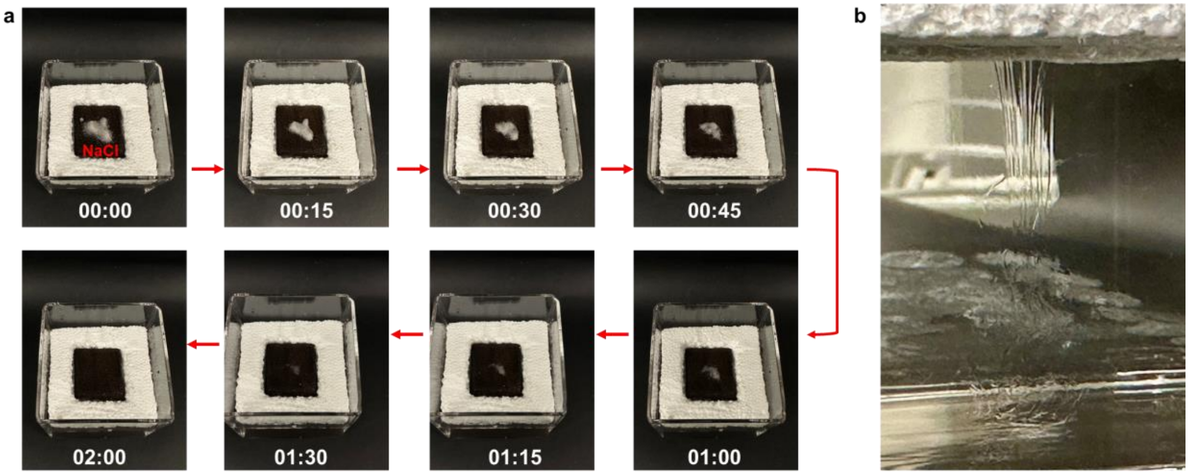

Supplementary Figure 15. a. Illustration of the salt redissolving procedure with additional 1gram sodium chloride of p-SDWE under 1KW m^{-2} illumination, **b.** Salt backflow driven by gradient and gravity

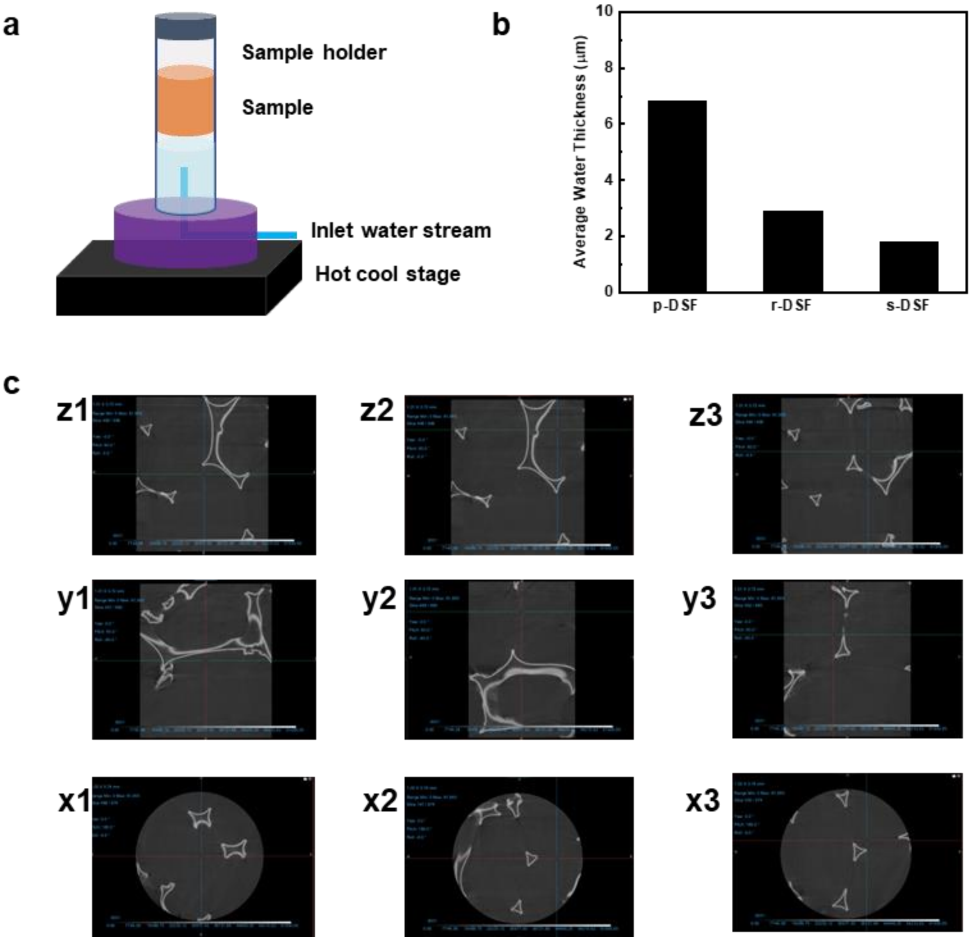

Supplementary Figure 16. a. Thin water layer characterization conducted by Micro-CT, **b.** Water thickness within s-SDWE, r-SDWE and p-SDWE, **c.** Cross view of water layer observed in p-SDWE

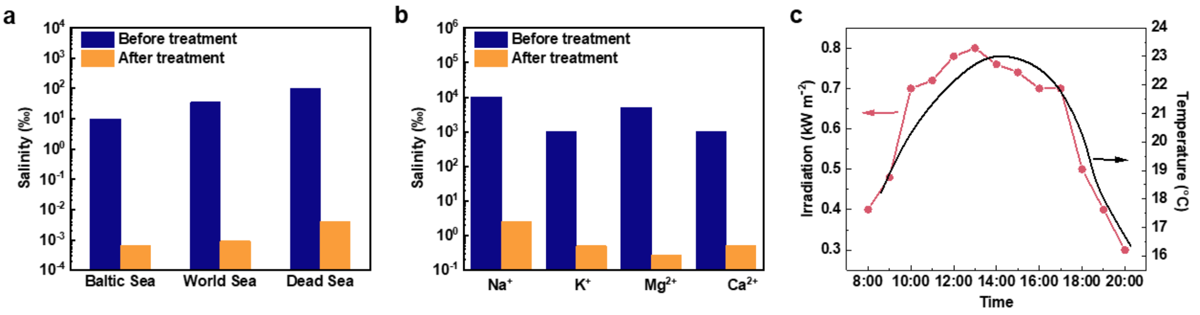

Supplementary Figure 17. a. The salinities of three artificial seawater samples before and after desalination using p-SDWE. **b.** Measured concentrations of four primary ions in an actual seawater sample before and after desalination, **c.** Solar radiation recorded over time on a sunny day from 08:00 to 20:00.

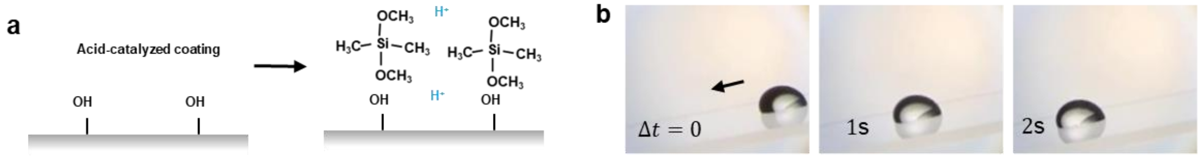

Supplementary Figure 18. a. Preparation of omniphobic liquid-like coating using acid-catalyzed method, **b.** Time-sequence images illustrating water slide on the omniphobic liquid-like coated glass substrate at a tilted angle of 10°.

Supplementary Table 1. Equivalent evaporation Enthalpy of Water and SDWEs

enthalpy (J g^{-1})	s-DSF	r-DSF	p-DSF	water
DSC	1610	1780	1930	2420
vaporization test	1032	1050	1080	

Supplementary Table 1 summarizes the total mass, mass of water, and calculated vaporization enthalpy of bulk water and our as-prepared samples. The measured enthalpy of water is 2420 J g^{-1}, which closely to the theoretical value of 2450 J g^{-1}, indicating the accuracy of our measurements. The vaporization enthalpy of water within the p-DSF was significantly lower than that of pure water. To determine the vaporization enthalpy, the evaporation enthalpy of water within DSF was measured. Specifically, water and DSF samples with the same surface area were placed together in a closed container along with a supersaturated potassium carbonate solution, creating a stabilized relative humidity (RH) under room temperature. The equivalent evaporation enthalpy (h_{eq}) of water in SDWEs can be estimated by vaporizing water with the same power input (U_{in}), using the equation (1):

$U_{in}= h_{eq}\ m_0= h_{vap}m_g$

where h_{vap} was the evaporation enthalpy and m_0 was the mass change of bulk water, while m_g referred to the mass change of the HNG sample. The evaporation rate of water and the corresponding calculated h_{eq}, where h_{eq} was 1930 J g^{-1} for p-DSF, was found to be significantly reduced compared to bulk water.

Supplementary Table 2. A summary of reported evaporators, varying in structural design, fluidic flow principles, cycling performance, resistance to salt fouling, and evaporation rate.

Evaporator's structure	Fluidic flow	Salt removal methods	Cycling operation time	Evaporation rate ($kg\ m^{-2}\ h^{-1}$)	Solar-to-steam efficiency	Net interfacial evaporation rate ($kg\ m^{-2}\ h^{-1}$)	References
Metallic λ-Ti3O5 nanoparticles-hydrogel	Concave cavity-induced flow	Salt reject （3.5wt%)	100 h	1.64 (2D evaporator) 6.09 (3D evaporator)	68.3% 95.9%	1.42 (2D evaporator) 4.31 (3D evaporator)	4
TEMPO-doped PDA-cellulose membrane	Unidirectional flow		1h*30	1.53	88.6%	1.362	5
2D Alumina sheet with aligned wicking tube	Vertical flow along the tube/microchannel	Open capillary tubes/reclean		1.26		0.62	6
Transformable 2D–3D	Directional	Light off wash		2.34 (2D evaporator) 4.35 (3D evaporator)		0.69 (2D evaporator) 1.34 (3D evaporator)	7
3D hydrogel	Unidirectional pump to the top surface		28 days	3.2	94%	2.9	8
3D sponge	Unidirectional flow with ultra-thin water supply	Day-night wash		3.2	91.6%	1.4	9
3D graphene foam with vertically oriented channel	Injection control water amount	Salt-resistant	100 h	2.40	99.4%	2.67	10
Multi-stage combined PV	Horizontal transport	Salt out-Salt crystallized at specific sites		2.45		1.77	11
Janus-interface solar-steam	Separated water and vapor			2.21	88%	1.95	12
Foam particles and aligned water channels	Directional flow	Salt resistant	100 h	2.25	136.7%	1.14	13
Wick-free confined water layer	Convective flow	Salt reject （20wt%)		1.36	91%	1.36	14
Contactless	Non contact	No salt (3.5wt%)		2.5	25%	2.5	15
Conical	Non-contact	Salt transport to specific area and recovery	40 h	1.25	89.9 %	1.25	16
Water evaporation surface and light absorption surface are physically separated	Directional flow	Salt crystallized at specific sites	288 h	2.42	94.3%	2.12	17
Asymmetric grooves and microcavity arrays	Marangoni flow	Salt crystallized at specific sites	9 h*10	2.63	96%	2.28	18
Thermo-responsive hydrogel	Unidirectional flow	Salt-free	10 h*7	4.145		N/A	19
Ammonia-Responsive +photocatalytic	Unidirectional flow		100 h	2.9 (2 sun)	97.3%	N/A	20
**Bilayer-structured thermo-responsive foam**	**Dynamic fluidic flow (thin water at evaporation transition to bulk water at salt self-washing)**	**Self washing**	**216 h**	**3.58**	**93.9%**	**3.13**	**This work**

Supplementary References

1. Owens D K, Wendt R C. Estimation of the surface free energy of polymers[J]. Journal of applied polymer science, 1969, 13(8): 1741-1747.
2. Girifalco L A, Good R J. A theory for the estimation of surface and interfacial energies. I. Derivation and application to interfacial tension[J]. The Journal of Physical Chemistry, 1957, 61(7): 904-909.
3. Ström G, Fredriksson M, Stenius P E R. Contact angles, work of adhesion, and interfacial tensions at a dissolving hydrocarbon surface[J]. Journal of colloid and interface science, 1987, 119(2): 352-361.
4. Yang B, Zhang Z, Liu P, et al. Flatband λ-Ti3O5 towards extraordinary solar steam generation[J]. Nature, 2023, 622(7983): 499-506.
5. Zou Y, Chen X, Yang P, et al. Regulating the absorption spectrum of polydopamine[J]. Science advances, 2020, 6(36): eabb4696.
6. Singh S C, ElKabbash M, Li Z, et al. Solar-trackable super-wicking black metal panel for photothermal water sanitation[J]. Nature Sustainability, 2020, 3(11): 938-946.
7. Wang Y, Wu X, Gao T, et al. Same materials, bigger output: A reversibly transformable 2D–3D photothermal evaporator for highly efficient solar steam generation[J]. Nano Energy, 2021, 79: 105477.
8. Zhao F, Zhou X, Shi Y, et al. Highly efficient solar vapour generation via hierarchically nanostructured gels[J]. Nature nanotechnology, 2018, 13(6): 489-495.
9. Wang Z, Wu X, He F, et al. Confinement capillarity of thin coating for boosting solar-driven water evaporation[J]. Advanced Functional Materials, 2021, 31(22): 2011114.
10. Liang H, Liao Q, Chen N, et al. Thermal efficiency of solar steam generation approaching 100% through capillary water transport[J]. Angewandte Chemie International Edition, 2019, 58(52): 19041-19046.
11. Wang W, Aleid S, Shi Y, et al. Integrated solar-driven PV cooling and seawater desalination with zero liquid discharge[J]. Joule, 2021, 5(7): 1873-1887.
12. Yao H, Zhang P, Yang C, et al. Janus-interface engineering boosting solar steam towards high-efficiency water collection[J]. Energy & Environmental Science, 2021, 14(10): 5330-5338.

13. Liu H, Chen B, Chen Y, et al. Bioinspired Self-standing, Self-floating 3D Solar Evaporators Breaking the Trade-off between Salt Cycle and Heat Localization for Continuous Seawater Desalination[J]. Advanced Materials, 2023, 35(24): 2301596.
14. Zhang L, Li X, Zhong Y, et al. Highly efficient and salt rejecting solar evaporation via a wick-free confined water layer[J]. Nature Communications, 2022, 13(1): 849.
15. Cooper T A, Zandavi S H, Ni G W, et al. Contactless steam generation and superheating under one sun illumination[J]. Nature communications, 2018, 9(1): 5086.
16. Wang L, Liu Z, Xu J, et al. Conical solar-thermo-radiative evaporator for sustainable desalination and salt recovery[J]. Desalination, 2023, 567: 116993.
17. Zhang C, Shi Y, Shi L, et al. Designing a next generation solar crystallizer for real seawater brine treatment with zero liquid discharge[J]. Nature Communications, 2021, 12(1): 998.
18. Wu L, Dong Z, Cai Z, et al. Highly efficient three-dimensional solar evaporator for high salinity desalination by localized crystallization[J]. Nature communications, 2020, 11(1): 521.
19. Lim H W, Park S H, Lee S J. 3D thermos-responsive hydrogel with enhanced water uptake and active evaporation for effective interfacial solar steam generation[J]. Desalination, 2023, 550: 116368.
20. Xing J, Tong J, Liu Y, et al. A high-efficiency ammonia-responsive solar evaporator[J]. Nanoscale, 2020, 12(17): 9680-9687.
