# Supplementary material for: Thermo-adaptive interfacial solar evaporation enhanced by dynamic water gating
Source: Nat Commun. 2024 Jul 22;15:6157. doi: 10.1038/s41467-024-50279-z (PMC11263690; doi:10.1038/s41467-024-50279-z)
Supplement: Supplementary file 3 — Description of Additional Supplementary Files [file 41467_2024_50279_MOESM3_ESM.pdf]

Description of Additional Supplementary Files

File Name: Supplementary Movie 1
Description: Thin Water Supply within PDA-assembled Microchannel (below LCST).

File Name: Supplementary Movie 2
Description: Bulk Water Flow and Saturated in the Whole Porous Structure (above LCSTfocused).

File Name: Supplementary Movie 3
Description: Salt Dissolving and Backflow assisted by thermo-responsive layer.

File Name: Supplementary Movie 4
Description: Micro-CT Visualization of Water Layer within p-SDWE.
